# Development and Initial Evaluation of Web-Based Cognitive Behavioral Therapy for Insomnia in Rural Family Caregivers of People With Dementia (NiteCAPP): Mixed Methods Study

**DOI:** 10.2196/45859

**Published:** 2023-08-24

**Authors:** Christina S McCrae, Ashley F Curtis, Melanie A Stearns, Neetu Nair, Mojgan Golzy, Joel I Shenker, David Q Beversdorf, Amelia Cottle, Meredeth A Rowe

**Affiliations:** 1 Department of Psychiatry University of Missouri Columbia, MO United States; 2 College of Nursing University of South Florida Tampa, FL United States; 3 Department of Family and Community Medicine University of Missouri Columbia, MO United States; 4 Department of Neurology University of Missouri Columbia, MO United States; 5 Departments of Radiology University of Missouri Columbia, MO United States; 6 Department of Psychological Sciences University of Missouri Columbia, MO United States; 7 The Thompson Center for Autism and Neurodevelopmental Disorders University of Missouri Columbia, MO United States

**Keywords:** arousal, caregivers, cognitive behavioral therapy, CBT: cognitive behavioral therapy for insomnia, CBT-I, dementia, insomnia, internet

## Abstract

**Background:**

Informal caregivers of people with dementia frequently experience chronic insomnia, contributing to stress and poor health outcomes. Rural caregivers are particularly vulnerable but have limited access to cognitive behavioral therapy for insomnia (CBT-I), a recommended frontline treatment for chronic insomnia. Web-based delivery promises to improve insomnia, particularly for rural caregivers who have limited access to traditional in-person treatments. Our team translated an efficacious 4-session standard CBT-I content protocol into digital format to create NiteCAPP.

**Objective:**

This study aimed to (1) adapt NiteCAPP for dementia caregivers to create NiteCAPP CARES, a tailored digital format with standard CBT-I content plus caregiver-focused modifications; (2) conduct usability testing and evaluate acceptability of NiteCAPP CARES’ content and features; and (3) pilot-test the adapted intervention to evaluate feasibility and preliminary effects on sleep and related health outcomes.

**Methods:**

We followed Medical Research Council recommendations for evaluating complex medical interventions to explore user needs and adapt and validate content using a stepwise approach: (1) a rural dementia caregiver (n=5) and primary care provider (n=5) advisory panel gave feedback that was used to adapt NiteCAPP; (2) caregiver (n=5) and primary care provider (n=7) focus groups reviewed the newly adapted NiteCAPP CARES and provided feedback that guided further adaptations; and (3) NiteCAPP CARES was pilot-tested in caregivers (n=5) for feasibility and to establish preliminary effects. Self-report usability measures were collected following intervention. Before and after treatment, 14 daily electronic sleep diaries and questionnaires were collected to evaluate arousal, health, mood, burden, subjective cognition, and interpersonal processes.

**Results:**

The stepped approach provided user and expert feedback on satisfaction, usefulness, and content, resulting in a new digital CBT-I tailored for rural dementia caregivers: NiteCAPP CARES. The advisory panel recommended streamlining content, eliminating jargon, and including caregiver-focused content. Focus groups gave NiteCAPP CARES high usefulness ratings (mean score 4.4, SD 0.79, scored from 1=least to 5=most favorable; score range 4.2-4.8). Multiple features were evaluated positively, including the intervention’s comprehensive and engaging information, caregiver focus, good layout, easy-to-access intervention material, and easy-to-understand sleep graphs. Suggestions for improvement included the provision of day and night viewing options, collapsible text, font size options, tabbed access to videos, and a glossary of terms. Pilot-test users rated usefulness (mean score 4.3, SD 0.83; range 4.1-4.5) and satisfaction (mean score 8.4, SD 1.41, scored from 1=least to 10=most satisfied; range 7.4-9.0) highly. Preliminary effects on caregiver sleep, arousal, health, mood, burden, cognition, and interpersonal processes (all *P*<.05) were promising.

**Conclusions:**

Adaptations made to standard digital CBT-I created a feasible, tailored digital intervention for rural dementia caregivers. Important next steps include further examination of feasibility and efficacy in a randomized controlled trial with an active control condition, a multisite effectiveness trial, and eventual broad dissemination.

**Trial Registration:**

ClinicalTrials.gov NCT04632628; https://clinicaltrials.gov/ct2/show/NCT04632628

## Introduction

### Overview

Approximately 16 million Americans serve as informal caregivers, providing 18.5 billion hours of care, which translates into US $234 billion in health care savings [1]. Dementia caregiving in particular is more demanding than other types of caregiving, due in part to time-consuming and unpredictable caregiving demands and associated emotional and physical exhaustion [1,2]. Informal caregivers of people with dementia frequently experience insomnia [3,4]. The number of people with dementia in the United States is projected to rise from 5.7 million to more than 14 million in the next 30 years [5]. Most people with dementia (70%) are cared for at home by a family member [1]. Thus, there is a critical need to evaluate the health of dementia caregivers and provide treatment for prevalent medical conditions such as insomnia.

In addition, because rural areas are aging faster than urban areas, the proportion of people at risk for dementia is growing faster, which translates to faster growth in the proportion of rural caregivers [6]. Compared to their urban counterparts, rural caregivers face additional challenges, including more difficulty accessing health care, a greater likelihood of social isolation, and increased vulnerability to sleep problems, stress, and depression. Rural caregivers have less access to care due to significant shortages of both primary and specialty care providers in rural areas [7,8] and are also at disproportionate risk for poverty and unemployment [2], which further limits their access. On average, they travel 144 km roundtrip to see their nearest health care provider [9]. Lengthy travel combined with the lack of public transportation in rural areas represent an additional major barrier to access. Thus, developing and evaluating treatments for medical conditions that are prevalent in caregivers (eg, insomnia) that can be accessed remotely (eg, on the internet) is a critical and currently unmet need in the health care community.

Research from our group and others verifies that caregivers have poorer sleep quality [10,11], take longer to fall asleep [10], spend more time awake during the night [3], and sleep less [10,12] than same-aged noncaregivers. Chronic insomnia (more than 3 months of difficulty initiating or maintaining sleep, early morning awakening, or nonrestorative sleep) affects up to 63% of caregivers [3,13] and tends to endure (18 years on average in our previous caregiver trial) [14,15]. Factors associated with caregiver insomnia include age-related sleep architecture changes, increased sympathetic nervous system arousal [3,15], increased vigilance [3], stress, depression, cognitive dysfunction, increased dementia risk, and person-related dementia behaviors (including nighttime wandering).

Cognitive behavioral therapy for insomnia (CBT-I) improves sleep (moderate or large effects) and mood (large effects) in adults of all ages [16] and is recommended by the American College of Physicians as a first-line treatment for chronic insomnia [17]. While research [18-20] shows that CBT-I is an efficacious treatment for the chronic insomnia experienced by caregivers, rural caregivers face particular difficulties accessing behavioral treatments, which require specially trained providers and administration over multiple sessions in a provider’s office. In a recent randomized controlled trial, our group found that brief CBT-I delivered through telehealth (videoconferencing) reduced caregiver sleep onset latency compared to sleep hygiene education in caregivers. Our CBT-I protocol was adapted with permission from McCurry and colleagues’ [3] earlier version [21] and included standard CBT-I techniques (sleep hygiene, stimulus control, and cognitive therapy), modified CBT-I techniques (sleep compression replaces sleep restriction, which is potentially burdensome for caregivers due to temporary sleep deprivation; brief hybrid relaxation is included given caregiver time constraints [22]; and muscle tensing is excluded given potential pain or musculoskeletal issues in older caregivers), additional techniques for caregivers (problem-solving, as skill deficiency can contribute to caregiver insomnia [23,24]), and stress management (respite, staying healthy, and communication). Our findings indicate CBT-I tailored for caregivers translates well to telehealth delivery and that technology holds great potential to increase rural caregiver access to behavioral interventions. However, telehealth delivery still requires considerable time commitment from trained therapists (who are already in short supply).

A small but growing body of evidence shows that web interventions have been successfully implemented in rural areas using the internet and other mobile platforms and improved noninsomnia health outcomes in stroke and caregivers [25-32]. Moreover, web-based CBT-I has been implemented and found to be efficacious in noncaregiver patient populations. For example, Taylor et al [33] translated 6-week CBT-I to a web format and conducted a randomized controlled trial in military personnel (N=100; n=33 received web CBT-I, n=34 received in-person CBT-I, and n=33 received minimal contact control). Compared to control, web-based and in-person CBT-I improved sleep efficiency, sleep onset latency, and wake time after sleep onset (effects moderate for web-based, large for in-person). Internet CBT-I also improved insomnia severity and decreased sleep-related cognitive arousal [33]. Another 6-week web-based CBT-I program (SHUTi) [34] was shown to improve insomnia severity (as measured through the insomnia severity index) relative to a no-contact control (large effect size) in adults with insomnia. Noted limitations of internet-based treatments include a lack of personal contact to address treatment questions or concerns without a face-to-face or telephone component. Additionally, personal support or guided delivery has been associated with larger treatment effects for both in-person and web-based CBT-I [26]. Taken together, these findings provide support for the development of web-based CBT-I for caregivers and suggest personal support or guided delivery is important to maximize treatment adherence and efficacy. Thus, our team developed a web-based CBT-I for caregivers through an iterative process.

### Iterative Development Process

**Figure 1:**
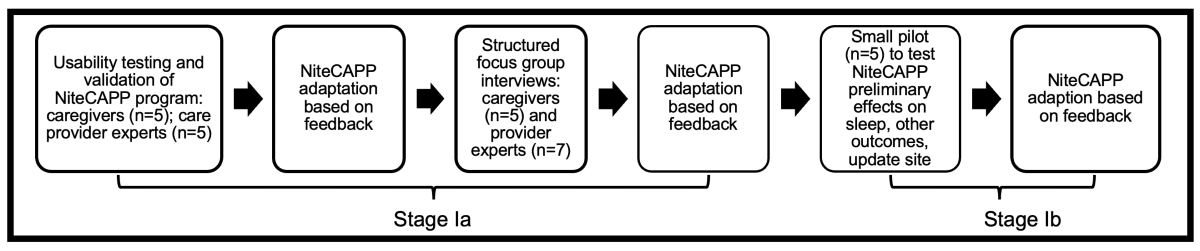
NiteCAPP usability testing, content evaluation, adaptation, and efficacy.

Following the Medical Research Council recommendations [35] for the evaluation of complex medical interventions, we conducted several steps in the initial development, pilot-testing, and evaluation of NiteCAPP CARES [36] (see Figure 1).

This paper describes stages 1a and 1b, which were conducted from 2019 to 2020. The goal of stage 1a was to translate (through feedback from caregivers and primary care providers; PCPs) an efficacious 4-session CBT-I into a web-based “NiteCAPP CARES,” a web-based behavioral treatment for insomnia for dementia caregivers that incorporates guided delivery through weekly therapist moderator feedback. A second goal of stage 1a was to conduct NiteCAPP CARES usability testing to evaluate the acceptability of its content and features. The goal of stage 1b was to pilot-test NiteCAPP CARES on several key outcomes (feasibility, favorability, satisfaction, sleep, arousal, mood, burden, and cognition) in a small sample of dementia caregivers. We hypothesized that NiteCAPP CARES would produce high ratings regarding favorability of program content. Further, we hypothesized that in the pilot study, participants would complete all 4 sessions on average and that satisfaction ratings would be at least more than 7 of 10 (highest) and usefulness ratings would be at least more than 4 of 5 (highest). Although the single-arm design of this pilot study precludes examination of efficacy, within-group effects of the treatment on sleep and related behaviors were also examined. We hypothesized that subjective sleep would improve immediately following treatment using NiteCAPP CARES. We further hypothesized that daytime functioning (ie, mood, caregiving burden, perceived stress, and cognition) would improve.

## Methods

### Stage 1a: Explore Stakeholder Views and Focus Groups

#### Participants

Purposeful criterion-i sampling, a nonrandom sampling method where individuals are selected because they have expertise or experience related to the study purpose, was used for both the advisory panel and focus group [37-39].

#### Advisory Panel

Stage 1a usability testing and validation of NiteCAPP content was first conducted by our web-based advisory panel of dementia caregivers (n=5; 3 of which were part of our community advisory board) and dementia experts, or PCPs (n=5; 2 of which are also part of our community advisory board). Participants were recruited through University of Missouri clinics, other providers, and the participant recruiter (AC). These individuals tested the NiteCAPP website on their own computers, tablets, or phones in their homes and then completed a modified structured internet intervention usefulness questionnaire (modified from existing surveys [34,40] as well as other studies evaluating web-based behavioral interventions [27,41,42]) to provide ratings of program content (1=least favorable to 5=most favorable) regarding ease of use, amount of information, website maintaining interest, adequate font size, videos maintaining interest, easy to understand, and helpful. Participants in the advisory panel also provided open-ended verbal and written feedback.

#### Focus Group

**Figure 2:**
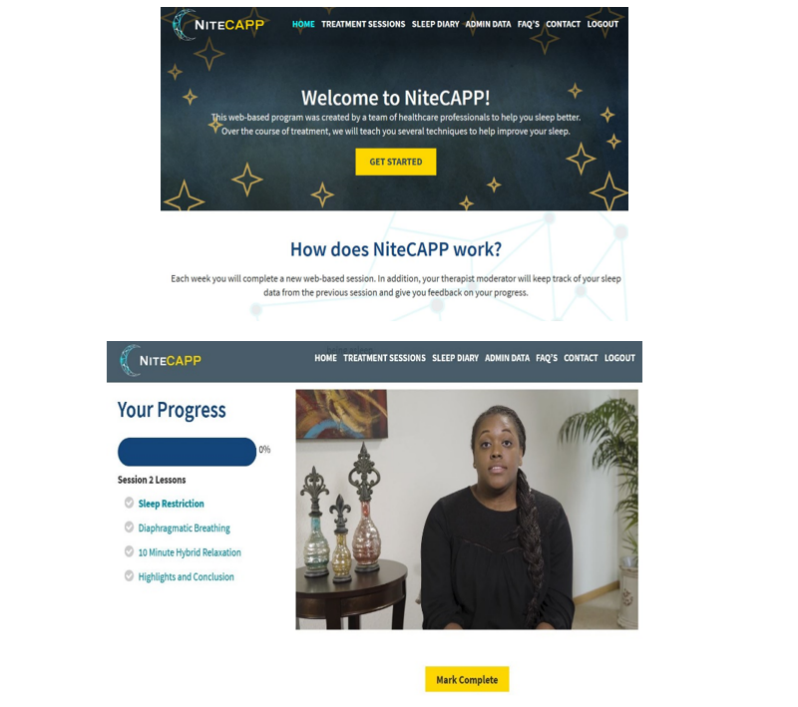
NiteCAPP CARES website home page.

Next, CSM (primary investigator) and AC (community caregiver consultant) led in-person focus groups consisting of caregivers (n=5; mean age 51.4, SD 15.9 years, age range 23-59 years; 3 female, 1 male, and 1 nonbinary; 4 White and 1 multiracial) and PCPs (n=7; mean age 45.29, SD 15.01 years; age range 26-66 years; 5 female and 2 male; 6 White and 1 multiracial). Users were provided logins and could review the NiteCAPP website (see Figure 2) on their own devices before and during the focus group.

Participants were encouraged to explore the site freely, read content, and watch and listen to audiovisual materials before and during the focus group. CSM and AC had a list of specific questions to ask, such as the usability of the website for caregivers and how they felt about the amount of text or number of pictures and asked the questions from the internet intervention usefulness questionnaire and satisfaction questionnaire in an open-ended format. They also encouraged participants to provide open-ended verbal comments or feedback on NiteCAPP. The focus group lasted approximately 90 minutes. At the end of the focus group, participants were asked to fill out a quantitative version of the internet intervention usefulness questionnaire.

Participants’ open-ended verbal comments were transcribed, and content analyses were performed to identify recurrent themes by CSM and AFC. Frequent topics were categorized and merged into common themes during a consensus meeting of CSM and AFC. Participants also indicated whether they had at-home internet access, indicated their method of internet access, and provided open-ended feedback on NiteCAPP.

### Stage 1b: Testing Feasibility and Preliminary Effectiveness

#### Participants

Stage 1b development involved testing NiteCAPP CARES in a single-arm pre-post-intervention pilot study to determine feasibility. The sample included dementia caregivers who had insomnia (n=5; see Table 1). Participants were recruited through physician or investigator referral (AFC or DQB) from Columbia, Missouri, and the surrounding regions and compensated with US $40 in gift cards (US $15 at the beginning of the study and US $25 at the end of the study).

Inclusion criteria for caregivers were (1) age older than 18 years; (2) primary caregiver living with person with dementia; (3) able to read and understand English; (4) no prescribed or over-the-counter sleep medicines or stabilized for more than 6 weeks; and (5) insomnia diagnosis based on sleep complaints for more than 6 months; adequate opportunity and circumstances for sleep; and more than 1 of the following: difficulty falling asleep, staying asleep, or waking too early; daytime dysfunction (mood, cognitive, social, or occupational) due to insomnia; and Insomnia Severity Index score ≥10. Exclusion criteria for caregivers were (1) unable to consent; (2) sleep disorder other than insomnia (ie, sleep apnea); (3) bipolar or seizure disorder; (4) other major psychopathology except depression or anxiety (eg, suicidal ideation or intent or psychosis); (5) severe untreated psychiatric comorbidity; (6) psychotropic or other medications (eg, β-blockers) that alter sleep; and (7) nonpharmacological treatment for sleep or mood outside of the current trial.

**Table 1:** Pilot study caregiver participant demographics (n=5).

Characteristics			Values
Age (years), mean (SD)			62.4 (18.2)
Age range (years)			33-78
Female, n (%)			3 (60)
**Relation to person with dementia, n (%)**			
	Spouse	4 (80)	
	Daughter	1 (20)	
Comorbid medical conditions, mean (SD)			3.2 (1.8)

#### Procedures

Participants were contacted through referral (AFC and DQB) and provided web-based documentation of informed consent. They were then asked to fill out a web-based screening form, including demographic information and medical history. If they qualified for the study, they filled out baseline questionnaires and electronic sleep diaries (see Measures section) and were then given access to NiteCAPP and completed the web-based 50 to 60–minute treatment sessions once per week for 4 weeks. They continued to fill out electronic daily sleep diaries throughout treatment to guide sleep prescription recommendations during treatment. Posttreatment assessment occurred immediately after treatment sessions ended and included questionnaires and daily diaries (for 2 weeks).

#### Intervention

A user-centered design approach was used and took into consideration age-related cognitive or perceptual concerns in our target population [43-47]. Interface features included (1) support for older adult readability [43,45] (eg, sans-serif typeface; font larger than 14 points; clear content headings; noncluttered backgrounds; brief videos; bright colors; visual contrast or ample white space; representative icons, such as a sleep diary icon with written diary on a bed; content enhancing, no distracting audio; clear navigation; and limited pull-down menus) and (2) ease of use (eg, simplified language, videos, pictographs, and guidance). Consistent with computer digital reminiscence and conversation aid recommendations [45], community member feedback was solicited on all aspects. Moderator feedback was provided to caregivers regarding their sleep and treatment progress. See Table 2 for session content.

**Table 2:** Session-by-session overview of NiteCAPP CARES.

Session number	
1	Sleep education, sleep hygiene, and stimulus control
2	Sleep compression, relaxation, and problem-solving
3	Coping, stress management, and cognitive therapy
4	Review, education, and planning for maintenance of behavior change

#### Measures

Measures were administered at baseline and immediately after treatment, unless otherwise specified.

#### Usability and Satisfaction

The modified internet intervention usefulness questionnaire (see Methods for stage 1a) was administered at the completion of NiteCAPP CARES. A proprietary 9-item satisfaction measure was also developed and administered at the completion of NiteCAPP CARES to obtain feedback on aspects of the study, including its structure, assessments within the NiteCAPP CARES site, scheduling, working with study staff, and whether they would recommend the study to others using a 10-point Likert scale (1=strongly disagree to 10=strongly agree).

#### Feasibility

Feasibility was assessed as the number of treatment modules completed (1-4, expressed as percentage) as well as the percentage of instructions followed (assessed through treatment adherence logs completed by participants within the session modules and confirmed by therapist moderators).

#### Sleep

Daily electronic diaries were completed for 14 days at baseline and immediately after treatment on the NiteCAPP CARES website and measured sleep onset latency (lights-out until sleep onset), wake after sleep onset, and sleep efficiency (total sleep time/time in bed×100%).

The Insomnia Severity Index [48] is a 7-item measure designed to assess the nature, severity, and impact of insomnia using a 5-point Likert scale, ranging from 0 (no problem) to 4 (very severe problem). Previous studies have shown excellent internal consistency (α=.91), convergent validity when compared to other measures (eg, fatigue), and that a cutoff score of 10 had 86.1% sensitivity and 87.7% specificity when determining insomnia [48].

#### Arousal

The Perceived Stress Scale [49] is a 10-item measure designed to assess past-month stress levels in response to everyday situations using a 4-point Likert scale, ranging from 0 (never) to 4 (very often). It has high reliability (α>.70) [50].

The Kingston Stress Scale [51] is 10-item measure designed to assess 3 categories: caregiving, family, and financial issues using a 5-point Likert scale, ranging from 1 (feeling fine or no stress) to 5 (extreme stress). It has high validity (α=.82) and reliability (α=.85) [51].

The Dysfunctional Beliefs and Attitudes about Sleep [52] is a 30-item measure designed to assess dysfunctional beliefs and attitudes about sleep using a 10-point Likert scale, ranging from 0 (strongly disagree) to 10 (strongly agree). Previous research has shown strong reliability (α=.77 for clinical and .79 for research samples) and temporal stability (*r*=0.83) [52].

#### Health and Mood

The Beck Depression Inventory-II [53] is a 21-item measure designed to assess depressive symptomatology using a 4-point Likert scale, ranging from 0 (absence of symptoms) to 3 (severe). Internal consistency is 0.90, and retest reliability ranges from 0.73 to 0.96 [53].

The State-Trait Anxiety Inventory [54] is a 20-item measure designed to assess anxiety using a 4-point Likert scale, ranging from 1 (not at all) to 4 (very much so). Reliability is high, with α=.93 for clinical and α=.92 for nonclinical samples [55].

#### Burden

The Zarit Burden Scale [56] is a 22-item measure designed to assess burden using a 5-point Likert scale, ranging from 0 (never) to 4 (nearly always). This measure has high reliability (α=.93) and convergent validity (*r*=0.53-0.73) [57].

The Dementia Patient’s Caregiver-Quality of Life [58] is a 20-item measure designed to assess how caregiver quality of life changes after beginning caregiving using yes-or-no questions and a 10-point sliding scale ranging from 0 (easy) to 10 (hard).

#### Cognition

Subjective cognition was measured using the Cognitive Failures Questionnaire [59], a 25-item measure designed to assess an individual’s perception of their own daily cognitive failures (eg, memory failures and distractibility) using a 5-point Likert scale, ranging from 0 (never) to 4 (very often). Higher scores indicate worse overall subjective cognition. Reliability is excellent, with α=.90 [60].

#### Qualitative Data

Participants were asked to provide qualitative feedback regarding how they felt about the written materials, audiovisual materials, and any comments or suggestions they felt would help to improve NiteCAPP CARES. Therapist moderator feedback was also solicited regarding suggestions to refine and improve the guided web-based support.

### Ethics Approval

This study was approved by the University of Missouri Institutional Review Board (2017125) and registered on ClinicalTrials.gov (NCT04632628).

## Results

### Stage 1a: Explore Stakeholder Views and Focus Groups

#### Advisory Panel

The advisory panel advised streamlining NiteCAPP content, eliminating jargon, and including caregiver-focused content.

#### Focus Group

For the focus group, average ratings for NiteCAPP features were high (see Table 3), with ratings ranging from 4.21 of 5 to 4.82 of 5 across all measured items.

Common themes included written material, audiovisual material, moderator feedback, and aesthetics of the website. Examples of comments on written materials included: “Easy to read and understand, but should have font size options,” “Great content but written at a pretty high literacy level,” and “Some sentences had too many clauses or more than one idea.” Focus group members stated that the audiovisual materials had “great information,” “were good...[and] engaging,” but should be “captioned or have a header.” NiteCAPP was then updated to incorporate suggestions for improvement based on common themes and titled NiteCAPP CARES (see Table 4). After that update, NiteCAPP CARES was pilot-tested in stage 1b.

**Table 3:** Stage 1a focus group usefulness ratings (n=5 dementia caregivers; n=5 dementia primary care providers). Scores based on a scale from 0 to 5, with higher scores indicating more favorable ratings.

Features		Mean score, (SD)	Minimum-Maximum score
**Website**			
	Ease of use	4.23 (1.40)	3-5
	Amount of info	4.40 (1.16)	2-5
	Maintains interest	4.24 (0.53)	4-5
	Font size	4.82 (0.79)	3-5
**Videos**			
	Maintain interest	4.21 (0.89)	3-5
	Easy to understand	4.40 (1.00)	3-5
	Helpful	4.44 (0.98)	3-5

**Table 4:** Stage 1a focus group feedback.

Feature	Themes			CG^a^ and PCP^b^ quotes
	Positive	Negative		
Written material	Thorough for target group Promotes independent completion of intervention Therapist moderator visual feedback helpful.	No font size options Excessive medical jargon Some instructions may elicit stress	“Easy to read and understand, but should have font size options.” [CG-001] “Information broken down into easy to digest chunks and not overwhelming to read through.” [CG-005] “I like the sense that one could complete these modules independently.” [PCP-001] “Minimize clinical/diagnostic terms.” [PCP-001] “I think incorporation of graphs and charts for sleep feedback is awesome.” [PCP-002] “Change the word ‘homework’ to something less stressful.” [CG-003]	
Audiovisual material	Rich, engaging information Important communication tool for target population Good pacing	Difficult to find on mobile	“Great information!” [CG-003] “The videos were good, and I found them to be engaging.” [PCP-004] “The videos feel a bit hidden, hard to find. Having a tab with all videos might be an option.” [CG-005] “I think videos are integral to the program. I think her pace is probably good for this patient population, since they will not have heard most of this before.” [PCP-002]	
Aesthetics	Good use of visual contrast	Lack of viewing options for daytime/nighttime	“Have a dark mode or night mode option, will help user’s eyes, especially if using at night.” [CG-005] “Bolding specific words and phrases helps make information accessible.” [CG-005]	
Navigation	Good layout and use of links/tabs to intervention material	Page content requires too much scrolling Too much text, should be hidden/collapsible	“Good links to other sessions and lessons.” [CG-001] “It’s a lot to navigate on mobile.” [CG-005] “Caregivers are exhausted and need things in small allotments...devices this will be viewed on are smaller in terms of landscape and how one moves beyond the initial screen.” [PCP-001] “Have collapsible tabs that can be expanded/collapsed when necessary to help people scrolling on their phone.” [CG-005] “I think the pages could also benefit from drop down menus to hide information and have text appear when the user clicks on it. It might make the pages less cluttered and feel more interactive.” [PCP-003]	
Resources		Lack of glossary	“Build in a glossary or a ‘terms to know’” [CG-003]	

^a^CG: caregiver.

^b^PCP: primary care provider.

### Stage 1b: Testing Feasibility and Preliminary Effectiveness

Feasibility and acceptability were excellent, with high average completion (>100% of sessions), adherence (76% for sleep hygiene, 80% for stimulus control, and 81% for relaxation based on daily logs), satisfaction (>8.4/10 on the Satisfaction Survey; see Table 5), and usefulness ratings (>4.3/5 on the Internet Intervention Utility Questionnaire; see Table 6). As shown in Table 7, NiteCAPP CARES led to improvements in subjective sleep (sleep onset latency, wake after sleep onset, sleep efficiency, and the Insomnia Severity Scale), arousal, mood (depression), burden, quality of life, perceived stress, and subjective cognition.

**Table 5:** Stage 1b pilot test satisfaction ratings (n=5 caregivers with insomnia). Scores based on a scale from 0 to 10, with higher scores indicating more favorable ratings.

	Mean score (SD)	Minimum-Maximum score
Expected experience	7.44 (3.98)	1-10
Scheduling convenience	9.03 (1.73)	7-10
Surveys and forms tolerable	8.49 (1.73)	7-10
Daily diaries tolerable	8.54 (1.41)	7-10
Therapist moderator	8.04 (1.41)	7-10
Recommend to a friend	9.05 (1.41)	7-10

**Table 6:** Stage 1b pilot test usefulness ratings (n=5 caregivers with insomnia). Scores were based on a scale from 0 to 5, with higher scores indicating more favorable ratings.

		Mean score (SD)	Minimum-Maximum score
**Website**			
	Ease of use	4.50 (0.83)	3-5
	Amount of info	4.33 (0.55)	4-5
	Maintains interest	4.29 (0.84)	3-5
	Font size	4.32 (0.50)	4-5
**Videos**			
	Maintain interest	4.13 (0.84)	3-5
	Easy to understand	4.40 (0.89)	3-5
	Helpful	4.18 (0.89)	3-5

Following this pilot test, additional participant qualitative feedback (see Table 8) was used to update NiteCAPP CARES a third time. Participants stated that the written and audiovisual materials were clear, though audiovisual materials were repetitive. Comments and suggestions included moving relaxation techniques to the morning and the importance of a schedule. Therapist moderator feedback was used to refine the guided web-based support and develop a moderator manual with clear, step-by-step instructions, schedule for administration of support, and case examples (see Textbox 1).

**Table 7:** Stage 1b pilot results. Caregiver subjective sleep, arousal, mood, burden, and cognition (n=5). Measures collected electronically.

Measure			Base				Post				Base to post						
			Mean (SD)		SE		Mean (SD)		SE		**Δ**		*t* test (*df*)		*P* value		Effect size^a^
**Subjective sleep**																	
	Sleep onset latency (min)	23.2 (8.67)		3.88		10.69 (4.34)		1.94		12.53		4.01 (4)		.01		1.46 (very large)	
	Wake after sleep onset (min)	54.95 (23.03)		10.30		22.30 (8.70)		3.89		32.65		4.05 (4)		.01		1.50 (very large)	
	Sleep efficiency^b^ (%)	79.64 (9.92)		4.44		92.67 (1.96)		0.88		13.03		3.53 (4)		.02		1.46 (very large)	
	Insomnia Severity Index^c^	15.20 (4.09)		1.82		8.00 (6.00)		2.68		7.20		2.25 (4)		.04		0.81 (large)	
**Subjective arousal**																	
	Perceived Stress Index	25.40 (5.50)		2.46		20.00 (8.97)		4.01		5.40		2.09 (4)		.05		0.58 (moderate)	
	Kingston Stress Scale	20.60 (3.65)		1.63		6.20 (2.95)		1.30		2.60		1.73 (4)		.08		0.63 (moderate)	
	DBAS^d^	130.80 (30.47)		13.63		77.40 (36.10)		16.14		53.40		3.87 (4)		.01		1.28 (large)	
**Health or mood**																	
	BDI-II^e^	11.80 (4.32)		1.93		6.20 (2.95)		1.32		5.60		2.89 (4)		.02		1.21 (large)	
	STAI^f^	61.40 (11.08)		4.96		57.20 (8.67)		3.88		4.20		1.36 (4)		.12		0.34 (small)	
**Burden**																	
	Zarit Burden Scale	23.40 (9.74)		4.35		17.20 (10.76)		4.81		6.20		2.16 (4)		.05		0.48 (small-moderate)	
	CG-QOL^g^	51.00 (19.81)		8.86		63.00 (14.40)		6.44		12.00		2.33 (4)		.04		0.55 (moderate)	
**Subjective cognition**																	
	CFQ^h^	43.80 (14.38)		6.43		39.20 (10.52)		4.71		4.60		2.44 (4)		.04		0.29 (small)	
**Interpersonal processes**																	
	PCFUS^i^	39.20 (13.52)		6.04		37.40 (16.88)		7.55		1.80		0.64 (4)		.28		0.09	

^a^Within-group effect sizes based on Hedges *g*_av_. Values of .20=small, .50=moderate, and .80=large.

^b^Sleep efficiency: ratio of time spent sleeping/time in bed×100%.

^c^Insomnia Severity Index: 0=none, 28=severe.

^d^DBAS: Dysfunctional Attitudes and Beliefs about Sleep.

^e^BDI-II: Beck Depression Inventory-II.

^f^STAI: State Trait Anxiety Inventory.

^g^CG-QOL: Dementia Patient’s Caregiver Quality of Life.

^h^CFQ: Cognitive Failures Questionnaire.

^i^PCFUS: Patient-Caregiver Functional Unit Scale.

**Table 8:** Stage 1b caregiver evaluation comments.

Feature	PCG^a^ quotes
Written material	“It was clear and easy to understand.” [PCG-01] “I think it is straight forward and easy to understand.” [PCG-04]
Audiovisual material	“They were clear but somewhat repetitive.” [PCG-02] “I think they are well done and easy to understand.” [PCG-04]
Comments or suggestions	“The questions about using the relaxation techniques that particular night should be moved to the a.m. questionnaire sleep diary in the morning.” [PCG-05] “As a caregiver for someone with dementia and a livestock owner. It’s difficult to maintain a set schedule.” [PCG-01] “This is a useful tool for a caregiver in conjunction with methods to get the care partner to sleep on a schedule.” [PCG-03]

^a^PCG: pilot-test caregiver.

**NiteCAPP CARES stage 1b pilot-test moderator feedback.**

**Moderator feedback to incorporate**

- A clear schedule for therapist moderator notes (eg, a set schedule of when to complete notes and send them to participants)
- A manual with templates for moderator notes for each stage of the study.
- Automated emails informing the moderator when a participant finishes a particular assignment
- Automated reminders for participants to complete diaries

## Discussion

### Overview

This study used mixed methods of qualitative and quantitative expert (PCPs) and participant-focused (rural dementia caregivers) feedback to iteratively adapt a 4-session web-based CBT-I (NiteCAPP) into a tailored digital format with standard CBT-I content plus caregiver-focused modifications (NiteCAPP CARES). The usability and acceptability of NiteCAPP CARES were then evaluated. The website was evaluated as easily accessible and useful, and the feasibility of the web-based intervention was high, suggesting that NiteCAPP CARES is highly functional for rural caregivers and PCPs. Preliminary efficacy testing also indicated that NiteCAPP CARES improved subjective sleep as well as several key aspects of daily functioning (eg, arousal, mood, burden, and subjective cognition).

Consistent with our hypotheses, the acceptability and usability of NiteCAPP CARES were high. In comparison to other in-person or telehealth versions of CBT-I [61-64], NiteCAPP CARES may offer unique advantages for treating insomnia, particularly in dementia caregivers. For example, NiteCAPP CARES provides greater caregiver accessibility given their demanding schedules since caregivers can access the materials on their schedule without having to make any appointments. NiteCAPP CARES is also less burdensome for both the patient and the therapist moderator, as trained behavioral sleep therapists who can conduct in-person or telehealth CBT-I are in short supply [8]. Further, compared to other web-based insomnia treatments [65-69], NiteCAPP CARES provides important enhancements for older caregivers. For example, although other 5 to 6–session, web-based CBT-I platforms [65,66] provide audiovisual content, NiteCAPP CARES provides tailored audio and visual recommendations for dementia caregivers. In addition, unlike other web-based CBT-I platforms, NiteCAPP CARES also provides a help forum to provide 24-7 assistance from therapist moderators. Other web-based CBT-I platforms [67] use sleep diaries and have a clinician and patient portal but function as adjuncts to in-person treatment, whereas NiteCAPP CARES is a standalone moderated treatment that allows the caregiver to access all the materials on their schedule. Other web-based CBT-I platforms [68,69] offer 6 sessions with tailored sleep recommendations based on either sleep diaries or screening questionnaires. One web-based CBT-I [69] even provides a web-based therapist who advises the patient on restructuring their cognitions, but this feedback does not extend to the patient’s sleep. However, none of these web-based insomnia treatments provide a simpler interface tailored for older caregivers or provide tailored and moderated therapy throughout treatment. In addition, the NiteCAPP CARES website itself was rated very highly in terms of its ease of use and readability (eg, font size), which were part of its initial user-centered design that took age-related cognitive or perceptual concerns in our target population into consideration (see Methods section) [70-74]. Second, caregiver use was likely promoted through the use of nonstructured session times (caregivers were encouraged to complete the sessions on their own schedule), simplified language, videos, and pictographs.

As expected, preliminary results also reveal that NiteCAPP CARES shows promise for improving the majority of our key outcomes. The moderate to large improvements across sleep outcomes (sleep onset latency, wake after sleep onset, sleep efficiency, and insomnia severity) found for NiteCAPP CARES are similar or greater than those found for other web-based CBT-I platforms [65,66,69]. Extending findings from previous web-based CBT-I interventions, our preliminary results also provide novel findings regarding NiteCAPP CARES’ potential impact on stress or arousal and subjective cognition (a treatment outcome that has not previously been assessed). NiteCAPP CARES showed improvements in subjective arousal (moderate to large effects) and cognition (small effects), as well as burden (small to moderate effects). Unlike other web- and app-based CBT-I platforms [65-69], the use of a therapist moderator was noted as a positive feature of the intervention during pilot-testing and likely contributed to its acceptance and high completion rate. Previous completion percentages of therapist-led CBT-I treatment range from 63% to 88% [61,62] for telehealth treatment and 80% to 98% [63,64] for in-person treatment, both below NiteCAPP CARES’ 100%. Similarly, average treatment adherence for therapist-led CBT-I is 38% and digital CBT-I is 35%, both lower than NiteCAPP CARES’ 76%-81% [75]. NiteCAPP CARES suggests that moderated web-based interventions may be comparable to or surpass the completion rates for in-person and telehealth delivery of CBT-I in this population. This will provide increased accessibility for caregivers living in rural areas that are not close to health care providers.

### Clinical Implications

Although further testing of NiteCAPP CARES is currently underway [76], these preliminary findings potentially suggest several clinical implications. Improving caregiver sleep is critical given that caregiver insomnia is associated with negative changes in central stress processing, resulting in poor physical health (altered heart rate and heart-rate variability) [77] and mood (anxiety and depression) [18,78]. Insomnia is also associated with poor caregiver cognition (processing speed [79,80], attention [79,81,82], executive functioning [82], and memory [82,83]) and neurodegenerative biomarkers associated with Alzheimer disease [83]. Poor mental and physical health makes it harder for caregivers to serve as caregivers and poor caregiver sleep contributes to early institutionalization [84]. Thus, treating caregivers’ insomnia may allow them to continue to provide care longer. Further, NiteCAPP CARES is a moderated, web-based intervention, as opposed to more traditional in-person or telehealth interventions that involve therapist-led sessions [85,86]. Thus, this intervention can be disseminated broadly, and the therapist moderator manual will enable other health care providers (eg, nurses and PCPs) to learn the treatment quickly and use NiteCAPP CARES in various treatment settings.

### Limitations and Future Directions

This study provides a foundation for future sleep research in rural dementia caregiver populations, which have largely been ignored. Our study has several limitations. First, the small number of individuals in our focus group and pilot test may not generalize to a broader population of caregivers and PCPs. Similarly, our small sample may have inflated our effect sizes; however, our previous studies in similar populations have shown these effect sizes to be robust [18,20,36]. Future studies should include a larger sample size that includes individuals from many different facets of life. For instance, it would be beneficial to gain insight from people with dementia, as NiteCAPP CARES treatment modules can be expanded to include resources specific to the care recipient. Furthermore, it would also be important to seek more evaluation from health care administration teams, who are likely to be the ones to give patients step-by-step instructions on how to log in and navigate NiteCAPP CARES. Future studies should focus on gaining feedback from dementia patient advocates, who have a considerable amount of insight on how to effectively care for and work with people with dementia and caregivers. The promising results from this preliminary development and initial evaluation study suggest a future randomized controlled trial that examines the efficacy of NiteCAPP CARES compared to an active web-based control in a larger sample of rural dementia caregivers is an important and warranted next step. Such future work may also benefit from a tailored, dyadic approach that involves the person with dementia in the treatment to the extent they are capable. Following further development and the establishment of efficacy, additional future steps include an effectiveness trial and broader implementation and dissemination efforts, as well as the development of a web-based, stand-alone sleep treatment for dementia caregivers. Specifically, as noted above, NiteCAPP CARES’s moderated web-based intervention provided excellent adherence and engagement compared to completion rates for other web-based treatments [58-61]. While this preliminary study’s results support our use of a moderated approach, a web-based, stand-alone option may be preferred by some caregivers and would provide them with an additional access option for obtaining CBT-I.

### Conclusions

A brief, web-based CBT-I platform called NiteCAPP CARES was created for caregivers to address caregiver sleep issues. Initial website feedback was accessed by asking rural caregivers and PCPs their opinions on NiteCAPP CARES. They thought the site was easy to understand, engaging, and well designed. Preliminary pilot-test data indicated that caregivers used the site (high treatment adherence and session completion), found it useful, and reported high satisfaction. NiteCAPP CARES also improved subjective sleep and daily functioning. These promising outcomes suggest that NiteCAPP CARES can be a beneficial sleep tool for both rural dementia caregivers and health care providers. Underserved populations who have difficulty obtaining evidence-based health care may particularly benefit from NiteCAPP CARES given its web-based delivery, which can be accessed anywhere the internet is available. A future randomized controlled trial to evaluate NiteCAPP CARES compared to an active web control in a larger sample is needed.

## Abbreviations

CBT-I: cognitive behavioral therapy for insomnia
PCP: primary care physician
